# Genotype and Phenotype Analysis of Chinese Children With Tuberous Sclerosis Complex: A Pediatric Cohort Study

**DOI:** 10.3389/fgene.2020.00204

**Published:** 2020-03-10

**Authors:** Yifeng Ding, Ji Wang, Shuizhen Zhou, Yuanfeng Zhou, Linmei Zhang, Lifei Yu, Yi Wang

**Affiliations:** Department of Neurology, Children’s Hospital of Fudan University, Shanghai, China

**Keywords:** tuberous sclerosis complex, genotype, phenotype, children, Chinese

## Abstract

Tuberous sclerosis complex (TSC) is a genetic condition characterized by the occurrence of hamartomatous wounds stemming from the dysfunction of the mammalian target of rapamycin (mTOR) pathway. We investigated the clinical phenotypes and genetic variants in 243 unrelated probands and their families in China. Exome sequencing, targeted sequencing or multiplex ligation-dependent probe amplification (MLPA) was performed in 174 children with TSC, among whom 31 (17.82%) patients/families were identified as having pathogenic or likely pathogenic variants in the *TSC1* gene, 120 (68.97%) as having pathogenic or likely pathogenic variants in the *TSC2* gene and 23 (13.21%) as having no pathogenic or likely pathogenic variants identified (NMI). In the 31 patients with pathogenic or likely pathogenic *TSC1* variants, 10 novel variants were detected among 26 different variants. In all 120 patients with *TSC2* variants, 39 novel variants were found among a total of 107 different variants. We compared the phenotypes of the individuals with *TSC1* pathogenic variants, *TSC2* pathogenic variants and NMI. Patients with *TSC2* variants were first diagnosed at a younger age (*p* = 0.003) and had more retinal hamartomas (*p* = 0.003) and facial angiofibromas (*p* = 0.027) (age ≥ 3 years) than individuals with *TSC1* variants. Compared with individuals with *TSC1/TSC2* pathogenic variants, NMI individuals had fewer cortical tubers (*p* = 0.003). Compared with individuals with *TSC1* pathogenic variants, NMI patients had more retinal hamartomas (*p* = 0.035), and compared with individuals with *TSC2* pathogenic variants, they had less epilepsy (*p* = 0.003) and fewer subependymal nodules (SENs) (*p* = 0.004).

## Introduction

As an autosomal dominant disease, tuberous sclerosis complex (TSC, OMIM #191100, #613254) is characterized by a wide range of disease severities involving hamartomatous tumors in multiple organs. The incidence of TSC is 1 in 6,000–10,000 live births, and it affects nearly 2 million individuals worldwide (Curatolo et al., 2008; Peron and Northrup, 2018). TSC is the result of mutations in either the *TSC1* (OMIM #605284) or *TSC2* (OMIM #191092) gene, triggering the hyperactivation of the mechanistic target of rapamycin (mTOR) signaling pathway, and subsequent cell proliferation deregulation (Cheadle et al., 2000; Crino et al., 2006; Henske et al., 2016; Franz and Krueger, 2018).

In this study, we combined the latest TSC gene testing with the clinical data of patients to evaluate the phenotype-genotype correlation.

## Materials and Methods

### Patients

A retrospective chart review was carried out at the Children’s Hospital of Fudan University. The review included children (age < 16 years) with TSC seen between August 2013 and July 2018. In total, 243 unrelated probands met the clinical diagnostic criteria for TSC (Northrup and Krueger, 2013). Exome sequencing or targeted sequencing was performed in 174 probands and their families. Our research was carried out in accordance with the Declaration of Helsinki and was approved by the Ethics Committee of the Fudan University Children’s Hospital. Signed informed consent was provided by the patients’ parents.

### Clinical Data

We studied the clinical information obtained from the medical records and phone calls with the families, including information on each patient’s birth history, family history, age at seizure inception, seizure forms, treatments, neurological development, central nervous system manifestations, renal disease, cutaneous manifestations, and tuberous sclerosis-associated neuropsychiatric disorders (TAND).

### Genetic Analysis

Extraction of genomic DNA from whole blood of patients and their patients was performed using the Agilent (Santa Clara, CA, United States) SureSelectXT Human All Exon 50 Mb kit according to the manufacturer’s instructions. *TSC1* and *TSC2* variants were detected in individuals by clinical exome sequencing (emphasis on 2742 genes) or whole-exome sequencing (WES). Our databank and public databases (the exome aggregation consortium, the 1000 Genome Project, and dbSNP137 reported in the UCSC Genome Browser) were utilized for variant screening. Candidate variants were verified using Sanger sequencing. Detection of significant deletions/repeats in TSC1 and TSC2 was performed using multiplexed-dependent probe amplification (MLPA) (MRC-Holland, Probemix *TSC2* P046, Probemix *TSC1* P124) following the manufacturer’s instructions. The variants were considered to be pathogenic on the basis of the following (Richards et al., 2015; Yang et al., 2018): (1) this variant would likely explain the indication for TSC and may be responsible for the clinical manifestation; (2) there is a null variant (non-sense, frameshift, canonical ± 1 or 2 splice sites, initiation codon, single or multiexon deletion) in the *TSC1/TSC2* gene causing a loss of function (LOF); or the same amino acid change as a previously established pathogenic variant regardless of nucleotide change is a known mechanism of TSC; and (3) the mutation is inherited from the affected parents or is *de novo* (both maternity and paternity confirmed) in the proband with a negative TSC family history.

### Statistical Analysis

Continuous variables are expressed as medians and ranges or as the means and standard deviations (SDs), while categorical data are presented as percentages and numbers where appropriate. TSC individuals were classified into the following three groups according to the results of the molecular analyses: variants in *TSC1*, variants in *TSC2*, and NMI. Then, we compared the clinical characteristics among the three groups using the Kruskal-Wallis tests for continuous variables and Fisher’s exact tests for categorical data. *P* values < 0.05 were considered statistically significant. Analyses were performed using Stata 15.1^1^.

## Results

### Cohort Characteristics

We identified and enrolled 174 unrelated patients with a definite clinical diagnosis of TSC. There were 88 (50.57%) females and 86 (49.43%) males. The median age at first presentation was 24.35 months (interquartile range: 9.56–68.83 months), and the median duration of follow-up was 28.81 months (interquartile range: 14.09–51.35 months). Thirty-one patients (17 males and 14 females; median age at first presentation: 34.79 months) had variants in the *TSC1* gene; 120 (58 males and 62 females; mean age at first presentation: 21.70 months) had variants in *TSC2* (*TSC2: TSC1* = 7.06:1); and 23 (11 males and 12 females; mean age at first presentation: 19.15 months) had NMI.

### Clinical Outcomes

Phenotype data regarding the major and minor diagnostic criteria for TSC and neurobehavioral features were collected from 174 affected individuals who met the criteria for a definite diagnosis of TSC. There are myriad neurologic sequelae of TSC, including neurodevelopmental disorders, epilepsy, and intellectual impairment, as well as many structural lesions. The most common manifestation leading to a diagnosis of TSC was the presence of epilepsy (140/174, 80.46%). During the follow-up period, a total of 148/174 (85.06%) patients suffered from epilepsy (*TSC1*:*TSC2*: NMI = 24:109:15), with 65.54% of those patients manifesting epilepsy within the first year of life. The seizure manifestations were variable, and 27.70% of patients presented with more than one seizure type. In our cohort, 44.59% (66/148) of the patients had infantile spasms, 52.03% (77/148) had focal seizures, and 33.11% (49/148) had another type of seizure. On average, patients were treated with ≥2 AEDs (median: 2; range: 0–7).

Compared with the general population, patients with TSC are at higher risk of developing neuropsychiatric disorders (de Vries et al., 2018). According to DSM-5, we defined all patients with IQ < 70 as intellectual disability (ID) patients, which accounted for 65.52% (114/174) of the population, including those with mild ID (IQ 51–70) and moderate ID (IQ 36–50), who accounted for 50% (87/174), and those with severe ID (IQ 20–35) and profound ID (IQ < 20), who accounted for 15.52% (27/174). In 97.70% (170/174) of the patients in this cohort, brain imaging abnormalities were found. Cortical tubers were found in up to 93.68% (163/174) of the patients and were located throughout all regions of the cortex and superficial white matter, especially the frontal lobe. As a major clinical feature of TSC, subependymal nodules (SENs) were found in up to 91.95% (160/174) of the patients, especially in the ependymal lining of the lateral ventricles. SEGA was diagnosed in 9/174 (5.17%) of the patients.

Retinal astrocytic hamartomas were detected in 38.89% (35/90) of the patients and were nearly universally benign.

In our cohort, 43.68% (76/174), 95.40% (166/174), 32.76% (57/174) and 3.05% (5/164) of TSC patients had facial angiofibromas, hypomelanotic macules, shagreen patches, and periungual fibroma, respectively. The presence of multiple facial angiofibromas in adolescence is characteristic of TSC.

Moreover, we found 61 patients with cardiac rhabdomyoma (35.06%) and 9 patients with liver hamartoma (5.17%).

### Genetic Analysis

In our study, 133 pathogenic or likely pathogenic variants were identified. Of these, 36.84% (49/133) were novel. Among all of the variants, most *TSC1* variants were truncating mutations, including non-sense mutations (38.71%) and frame shifts (29.03%), and most of the variants were located in crucial regions (N-terminal protein interaction domain and tuberin interaction coiled-coil domain). Most *TSC2* variants were missense mutations (24.17%), non-sense mutations (25.00%) and frame shifts (22.50%), and most of the variants were located in crucial regions (N-terminal hamartin interaction GTPase activation protein domain) (Table 1).

**TABLE 1:** Description of the types of mutations detected (*n* = 151).

	TSC1		TSC2	
Mutation type	n	%	n	%
Missense	5	16.13	29	24.17
Non-sense	12	38.71	30	25.00
Splicing	2	6.45	17	14.17
Frame shift	9	29.03	27	22.50
Small deletion	1	3.23	8	6.67
Large deletion	2	6.45	7	5.83
Deletion-insertion	0	0	2	1.66
Total	31	100.00	120	100.00

*TSC1, patients with TSC1 mutation; TSC2, patients with TSC2 mutation.*

A total of 31 patients (17.82%) had pathogenic variants or likely pathogenic variants of *TSC1*, 120 (68.97%) had pathogenic variants or likely pathogenic variants of *TSC2*, and 23 (13.21%) had NMI. In the *TSC1* group, we found 10 (39.29%) novel variants and 16 (60.71%) known variants. In our cohort, exon 15 of the *TSC1* gene had a relatively high probability of mutation (15/31, 48.39%). In the *TSC2* group, 39 novel pathogenic or likely pathogenic variants (36.45%) and 68 known variants (63.55%) were found. Among the 42 exons in the *TSC2* gene, exon 34 (13/120, 10.83%) and exon 41 (17/120, 14.17%) of the *TSC2* gene had relatively high mutational probabilities (Supplementary Table 1).

### Genotype-Phenotype Correlations in Patients With TSC

The main features of the three groups (*TSC1, TSC2*, and NMI) were compared in 174 patients who underwent complete genetic testing (point mutations and deletions/repetitions), as shown in Table 2. Compared with individuals with *TSC1* pathogenic variants, individuals with *TSC2* pathogenic variants were first diagnosed at a younger age (*p* = *0.003*) and had more retinal hamartomas (*p* = 0.003) and facial angiofibromas (*p* = 0.027) (age ≥ 3 years). Compared with individuals with *TSC1/TSC2* pathogenic or likely pathogenic variants, NMI individuals had fewer cortical tubers (*p* = 0.003). Compared with individuals with *TSC1* pathogenic of likely pathogenic variants, NMI individuals had more retinal hamartomas (*p* = 0.035), and compared with individuals with *TSC2* pathogenic of likely pathogenic variants, NMI individuals were less likely to have epilepsy (*p* = 0.003) and had fewer SENs (*p* = 0.004).

**TABLE 2:** Comparison of clinical manifestations among *TSC1*, *TSC2*, and NMI patients.

Phenotype	*TSC1*	*TSC2*	NMI	*p* value		
				*TSC1* vs. *TSC2*	*TSC1* vs. NMI	*TSC2* vs. NMI
*N*	31	120	23			
**Demographic characteristic**						
Male: female	17:14	58:62	11:12			
Family history of TSC	5/31	19/120	1/23	1.000	0.224	0.312
**Central nervous system**						
Epilepsy	24/31	109/120	15/23	0.059	0.369	0.003*
Age at seizure onset, months (median ± SD)	39.00 ± 45.12	14.10 ± 29.06	25.79 ± 35.37	0.003*	0.308	0.308
Seizure onset before age 1 year	9/24	81/109	8/15	0.001*	0.508	0.124
Spasm	10/24	51/109	5/15	0.821	0.496	0.173
SENs	27/31	112/120	21/23	0.304	0.204	0.004*
Cortical tubers	30/31	111/9	22/23	1.000	0.003*	0.013*
SEGA	0/31	7/120	2/23	0.347	0.186	0.645
Retinal hamartomas	1/15	28/59	6/14	0.003*	0.035*	1.000
**Cutaneous manifestation**						
Hypomelanotic macules	28/31	117/120	21/23	0.101	1.000	0.183
Facial angiofibromas (≥3 years)	9/27	56/94	7/17	0.027*	0.749	0.189
Shagreen patch	12/31	41/117	4/23	0.833	0.133	0.142
Periungual fibroma	0/30	5/111	0/23	0.580	Null	0.590
Cardiac rhabdomyoma	12/30	40/112	4/23	0.675	0.141	0.240
**Renal system**						
Multiple renal cyst	0/31	10/120	1/22	0.216	0.426	1.000
Renal angiomyolipoma	2/29	31/112	5/23	0.025*	0.219	0.796
**TAND**						
Intellectual disability	19/31	81/120	14/23	0.529	1.000	0.631
Autism	3/15	31/126	2/18	0.390	0.650	0.140

SENs, subependymal nodules; SEGA, subependymal giant cell astrocytoma; autism, autism spectrum disorder; *p < 0.05; Wilcoxon rank-sum tests for continuous variables, Fisher’s exact tests for categorical data.

After comparing multiple clinical phenotypes in individuals with different *TSC1/TSC2* mutation types, we found no significant differences in CNS, cutaneous manifestations, cardiac rhabdomyoma, renal system disorders, or TAND (Table 3).

**TABLE 3:** Clinical manifestations of different mutation types in TSC1 and TSC2 genes.

Phenotype	*TSC1*								*TSC2*							
	Mis	Non	Sp	Fs	Sd	Ld	Di	*p*	Mis	Non	Sp	Fs	Sd	Ld	Di	*p*
N	5	12	9	2	2	1	0		29	30	17	27	8	7	2	
**Central nervous system**																
Epilepsy	3 (60%)	8 (67%)	8 (89%)	2 (100%)	2 (100%)	1 (100%)	0 (0%)	0.720	26 (90%)	26 (87%)	16 (94%)	26 (96%)	8 (100%)	6 (86%)	1 (50%)	0.350
Spasm	0 (0%)	5 (63%)	2 (25%)	1 (50%)	1 (50%)	1 (100%)	0 (0%)	0.310	10 (38%)	14 (54%)	7 (44%)	12 (46%)	5 (63%)	3 (50%)	0 (0%)	0.850
SENs	4 (80%)	11 (92%)	8 (89%)	2 (100%)	1 (50%)	1 (100%)	0 (0%)	0.590	27 (93%)	29 (97%)	15 (88%)	26 (96%)	7 (88%)	6 (86%)	2 (100%)	0.590
Cortical tubers	4 (80%)	12 (100%)	9 (100%)	2 (100%)	2 (100%)	1 (100%)	0 (0%)	0.320	26 (90%)	30 (100%)	16 (94%)	24 (89%)	7 (88%)	6 (86%)	2 (100%)	0.360
SEGA	0 (0%)	0 (0%)	0 (0%)	0 (0%)	0 (0%)	0 (0%)	0 (0%)	Null	3 (10%)	3 (10%)	0 (0%)	0 (0%)	1 (13%)	0 (0%)	0 (0%)	0.370
Retinal hamartomas	0 (0%)	1 (13%)	0 (0%)	0 (0%)	0 (0%)	0 (0%)	0 (0%)	1.000	7 (54%)	5 (31%)	4 (44%)	7 (54%)	3 (60%)	2 (67%)	0 (0%)	0.740
**Cutaneous manifestation**																
Hypomelanotic macules	4 (80%)	11 (92%)	8 (89%)	2 (100%)	2 (100%)	1 (100%)	0 (0%)	0.870	28 (97%)	30 (100%)	17 (100%)	25 (93%)	8 (100%)	7 (100%)	2 (100%)	0.560
Facial angiofibromas	3 (60%)	5 (42%)	1 (11%)	1 (50%)	0 (0%)	0 (0%)	0 (0%)	0.310	10 (34%)	19 (63%)	10 (59%)	11 (41%)	3 (38%)	4 (57%)	2 (100%)	0.180
Shagreen patch	2 (40%)	4 (33%)	4 (44%)	2 (100%)	0 (0%)	0 (0%)	0 (0%)	0.510	9 (31%)	16 (55%)	6 (38%)	8 (31%)	1 (13%)	1 (14%)	0 (0%)	0.170
Periungual fibroma	0 (0%)	0 (0%)	0 (0%)	0 (0%)	0 (0%)	0 (0%)	0 (0%)	Null	2 (7%)	2 (7%)	1 (6%)	0 (0%)	0 (0%)	0 (0%)	0 (0%)	0.810
Cardiac rhabdomyoma	1 (20%)	4 (36%)	1 (11%)	2 (100%)	1 (50%)	0 (0%)	0 (0%)	0.190	10 (37%)	11 (41%)	8 (47%)	11 (41%)	3 (38%)	1 (17%)	0 (0%)	0.880
**Renal system**																
Multiple renal cyst	0 (0%)	0 (0%)	1 (11%)	0 (0%)	0 (0%)	0 (0%)	0 (0%)	0.620	4 (15%)	4 (15%)	2 (12%)	2 (7%)	2 (33%)	3 (50%)	0 (0%)	0.200
Renal angiomyolipoma	0 (0%)	1 (9%)	0 (0%)	1 (50%)	0 (0%)	0 (0%)	0 (0%)	0.330	6 (23%)	13 (48%)	3 (18%)	7 (26%)	1 (14%)	1 (17%)	0 (0%)	0.280
**TAND**																
Intellectual disability	2 (40%)	7 (58%)	8 (89%)	0 (0%)	1 (50%)	1 (100%)	0 (0%)	0.120	18 (62%)	19 (63%)	10 (59%)	22 (81%)	6 (75%)	5 (71%)	1 (50%)	0.600
Autism	0 (0%)	2 (40%)	1 (25%)	0 (0%)	0 (0%)	0 (0%)	0 (0%)	0.820	13 (52%)	5 (21%)	3 (23%)	7 (35%)	1 (20%)	3 (43%)	0 (0%)	0.290

*SENs, subependymal nodules; SEGA, subependymal giant cell astrocytoma; autism, autism spectrum disorder; Mis, Missense; Non, Non-sense; Sp, splicing; Fs, frame shift; Sd, small deletion; Ld, large deletion; Di, deletion-insertion. Fisher’s exact tests for categorical data.*

## Discussion

Tuberous sclerosis is a hereditary multisystem disease characterized by hamartomas in multiple organs, including the skin, brain, liver, kidneys, heart and eyes. After the discovery of the involvement of the *TSC1* and *TSC2* genes in TSC in 1997 and 1993, respectively, the genetic basis of the disease was studied to better understand its clinical manifestations. Unfortunately, despite the continuous development of new molecular detection techniques, understanding the complex clinical manifestations of TSC that predict the observed individual clinical phenotypes is still challenging. TSC1 and TSC2 encode hamartin and tuberin, respectively, which form heterodimers that act synergistically to regulate cell growth and proliferation (Plank et al., 1998; van Slegtenhorst et al., 1998; Han and Sahin, 2011). *TSC1* and *TSC2* variants are thought to result in a loss of function, leading to multiple organ hamartias and hamartomas (Au et al., 2004). In our cohort, all patients fulfilled the current diagnostic criteria for TSC. In total, 42.86% (6/14) of the *TSC1* variants were located in crucial regions (N-terminal protein interaction domain and tuberin interaction coiled-coil domain), and 51.35% (19/37) of the *TSC2* variants were located in crucial regions (N-terminal hamartin interaction GTPase activation protein domain). We compared the correlation between the clinical features obtained in our cohort with the different mutation types found in previous studies (Dabora et al., 2001; Sancak et al., 2005; Au et al., 2007; Yang et al., 2017).

As shown in Table 1, patients with *TSC2* variants were identified at a younger age (*p* = 0.003) and showed more retinal hamartomas and facial angiofibromas compared with patients with *TSC1* variants (age ≥ 3 years) (*p* = 0.027). NMI patients had fewer cortical tubers (*p* = 0.003) compared to patients with *TSC1/TSC2* variants. Compared with patients with *TSC1* variants, NMI patients exhibited more retinal hamartomas (*p* = 0.035), were less likely to have epilepsy (*p* = 0.003), and had fewer cortical tubers (*p* = 0.013) and SENs (*p* = 0.004) than patients with TSC2 variants.

As shown in Table 4, the frequency of ID (65.52% vs. 46.47∼82.41%), seizures (85.06% vs. 74.77∼96.58%) and SENs (91.95% vs. 83.39∼91.71%) in our cohort was consistent with that of previous studies. Compared with the cohorts in previous reports, our cohort may have more cortical tubers (93.68% vs. 83.97∼89.74%), retinal hamartomas (39.77% vs. 23.96∼37.68%) and hypomelanotic macules (95.40% vs. 89.10∼94.87%). Compared with reports from other countries, our TSC patients were more similar to those in the reports of Yang et al. Compared with patients in reports from other countries, our patients were less likely to develop SEGA (5.17∼9.40% vs. 11.06∼30.00%), facial angiofibromas (26.50∼43.68% vs. 60.06∼84.54%), shagreen patches (19.66∼33.33% vs. 38.94∼53.57%), multiple renal cysts (6.32∼14.53% vs. 24.79∼42.03%), and renal angiomyolipomas (23.01∼28.21% vs. 41.89∼54.11%), possibly due to the younger age of our cohort.

**TABLE 4:** Frequencies of major clinical characteristics in TSC patients in previous and current studies.

	*TSC1:* TSC2: NMI	ID	Epilepsy	SENs	Cortical tubers	SEGA	Retinal hamartomas	Hypomelanotic macules	Facial angiofibromas	Shagreen patch	Cardiac rhabdomyoma	Multiple renal cyst	Renal angiomyolipoma
Dabora et al., 2001	28:158:38	65.81 (102/155)	90.58 (202/223)	91.71 (177/193)	88.50 (100/113)	11.06 (23/208)	23.96 (46/192)	92.24 (202/219)	69.20 (155/224)	46.95 (100/213)	51.28 (100/195)	42.03 (87/207)	54.11 (112/207)
Sancak et al., 2005	53:182:27	82.41 (89/108)	90.91 (110/121)	90.32 (84/93)	88.89 (56/63)	30.00 (9/30)	37.68 (26/69)	92.16 (94/102)	84.54 (82/97)	53.57 (30/56)	39.66 (23/58)	32.84 (22/67)	41.89 (31/74)
Au et al., 2007	61:182:74	46.47 (112/241)	74.77 (243/325)	83.39 (231/277)	83.97 (220/262)	18.00 (45/250)	30.62 (64/209)	89.10 (286/321)	60.06 (191/318)	38.94 (118/303)	46.15 (114/247)	24.79 (60/242)	46.58 (109/234)
Yang et al., 2017	16:101	71.79 (84/117)	96.58 (113/117)	84.62 (99/117)	89.74 (105/117)	9.40 (11/117)	–	94.87 (111/117)	26.50 (31/117)	19.66 (23/117)	35.04 (41/117)	14.53 (17/117)	28.21 (33/117)
Present study	24:109:15	65.52 (114/174)	85.06 (148/174)	91.95 (160/174)	93.68 (163/174)	5.17 (9/174)	39.77 (35/88)	95.40 (166/174)	43.68 (76/174)	33.33 (57/171)	36.97 (61/165)	6.32 (11/174)	23.17 (38/164)

*ID, intellectual disability; TSC1, patients with TSC1 mutation; TSC2, patients with TSC2 mutation; SENs, subependymal nodules; SEGA, subependymal giant cell astrocytoma.*

This study has some limitations. Exome sequencing, targeted sequencing or MLPA was performed in 174 children. However, we have not performed further deep-depth sequencing of the 23 clinically confirmed NMI cases, nor have we sequenced tissue samples (such as brain tissue, etc.). The cases of somatic mosaicism are not yet clear. Assuming a somatic mosaicism detection rate of 5% (Kozlowski et al., 2007) and given that no mutation was identified in *TSC1* or *TSC2* by the sequence analyses in 13.21% of individuals with TSC, we conclude that fewer than 1% of persons with TSC have somatic mosaicism for a *TSC1* or *TSC2* mutation. Improving sequencing depth and collecting more tissue samples will lead to better somatic mosaicism detection.

As reported by Yang et al. (2017) despite these general associations, there is significant clinical variability among TSC patients, even within the same family. In our clinical experience, severe and mild cases can occur in patients with different genotypes; therefore, it is challenging to predict the clinical phenotype of TSC patients based on their genotype. Therefore, we recommend that clinicians use caution when counseling patients. For patients who receive a genetic diagnosis early in life, their genotypes should never be used to predict clinical outcomes unless there is an accurate genotype-phenotype correlation.

## Conclusion

TSC1 or TSC2 variants are key causes of TSC. The overall mutation rate of the TSC patients in our cohort was approximately 86–87%. TSC exhibits variability in clinical findings, with TSC2 variants producing a more severe phenotype than that observed in patients with TSC1 variants or NMI. Compared with TSC1 patients and NMI patients, those with TSC2 variants have a greater chance of developing renal abnormalities, intracranial lesions, and skin diseases, and their age at the onset of epilepsy is younger.

## Data Availability Statement

The genotyping data generated for this study can be found in the LOVD database (https://databases.lovd.nl/shared/variants?search_owned_by_=%3D%22Yifeng%20Ding%22), with associated accession numbers ranging from #0000642979 to #0000643105, #0000642877, #0000642883, #0000642972, #0000642973, #0000642974, and #0000642976. The whole exome sequencing data cannot be made publicly available because it is not allowed according to the national requirements about human genetic resource of research: The Regulations of the People’s Republic of China on the Management of Human Genetic Resources (http://www.moj.gov.cn/government_public/content/2019-06/10/593_236557.html). Requests to access the datasets should be directed to corresponding author.

## Ethics Statement

Our research was carried out in accordance with the Declaration of Helsinki and was approved by the Ethics Committee of the Fudan University Children’s Hospital. Written informed consent to participate in this study was provided by the participants’ legal guardian/next of kin.

## Author Contributions

YD and YW designed the research. YD, JW, SZ, YZ, LZ, and LY performed the collection and analysis of data. YW wrote the manuscript and approved the submitted version.

## Conflict of Interest

The authors declare that the research was conducted in the absence of any commercial or financial relationships that could be construed as a potential conflict of interest.

## Footnotes

1 www.stata.com

## Abbreviations

MLPA: multiplex ligation-dependent probe amplification
mTOR: mammalian target of rapamycin
NMI: no variants identified
SDs: standard deviations
SENs: subependymal nodules
TAND: tuberous sclerosis-associated neuropsychiatric disorders
TSC: tuberous sclerosis complex
WES: whole-exome sequencing.
