# Supplementary material for: Genotype and Phenotype Analysis of Chinese Children With Tuberous Sclerosis Complex: A Pediatric Cohort Study
Source: Front Genet. 2020 Mar 10;11:204. doi: 10.3389/fgene.2020.00204 (PMC7076134; doi:10.3389/fgene.2020.00204)
Supplement: Supplementary file 1 [file Table_1.docx]

Supplementary Table 1. General features of the patients with TSC1/TSC2 gene mutations

| No | Familial inheritance | Gender | Date Of Birth | Gene | Mutation type | EXON | Mutation site | Amino acid |
| --- | --- | --- | --- | --- | --- | --- | --- | --- |
| 1 | de novo | M | 2012/8/15 | TSC1 | Nonsense | 8 | c.682C>T | p.R228* |
| 2 | de novo | M | 2016/11/7 | TSC1 | Nonsense | 10 | c.959T>A | p.L320* |
| 3 | maternal | M | 2006/11/1 | TSC1 | Missense | 5 | c.278T>A | p.(Leu93Gln) |
| 4 | de novo | M | 2013/4/10 | TSC1 | Small deletion | 15 | c.1530-1531delCA | p.Asp510Glufs*24 |
| 5 | de novo | F | 2011/8/21 | TSC1 | Frame shift | 17 | c.2151delG | p.Arg717Serfs*7 |
| 6 | de novo | M | 2006/5/7 | TSC1 | Frame shift | 15 | c.1923-1929CTCTACC | p.S642Pfs*8 |
| 7 | de novo | F | 2005/3/26 | TSC1 | Nonsense | 18 | c.2356C>T | p.R786* |
| 8 | de novo | M | 2003/10/8 | TSC1 | Nonsense | 15 | c.1498C>T | p.Arg500* |
| 9 | de novo | F | 2011/8/29 | TSC1 | Nonsense | 15 | c.1498C>T | p.Arg500* |
| 10 | de novo | M | 2012/9/18 | TSC1 | Nonsense | 17 | c.2080C>T | p.Gln694* |
| 11 | de novo | M | 2004/7/31 | TSC1 | Missense | 23 | c.3177C>G | p.Phe1059Leu |
| 12 | de novo | M | 2013/3/4 | TSC1 | Frame shift | 15 | c.1756het_delT | p.C586Vfs*43 |
| 13 | de novo | M | 2012/2/5 | TSC1 | Large deletion | 15 | EX15del |  |
| 14 | maternal | F | 2012/7/26 | TSC1 | Missense | 15 | c.1960C>G | p.Gln654Glu |
| 15 | de novo | F | 2016/1/3 | TSC1 | Nonsense | 15 | c.1498C>T | p.Arg500* |
| 16 | maternal | M | 2010/7/7 | TSC1 | Missense | 10 | c.965T>C | p.Met322Thr |
| 17 | de novo | F | 2007/1/8 | TSC1 | Large deletion | 12 | EX12del |  |
| 18 | de novo | F | 2011/5/5 | TSC1 | Splicing | 10 | c.1029+1G>A |  |
| 19 | de novo | M | 2016/10/9 | TSC1 | Missense | 15 | c.1681C>G | p.Ser487Cys |
| 20 | paternal | F | 2013/1/25 | TSC1 | Frame shift | 11 | c.1084delC | p.P362Lfs*77 |
| 21 | de novo | M | 2015/2/16 | TSC1 | Frame shift | 15 | c.1904_1905delCA | p.Thr635Argfs*52 |
| 22 | de novo | M | 2010/11/16 | TSC1 | Frame shift | 15 | c.1888_1891delAAAG | p.Lys630Glnfs*22 |
| 23 | de novo | F | 2016/6/25 | TSC1 | Nonsense | 15 | c.1525C>T | p.R509* |
| 24 | paternal | F | 2015/3/7 | TSC1 | Splicing | 8 | c.737+1G>C |  |
| 25 | de novo | F | 2014/8/20 | TSC1 | Nonsense | 15 | c.1525C>T | p.R509* |
| 26 | de novo | F | 2006/1/20 | TSC1 | Nonsense | 8 | c.682C>T | p.Arg228* |
| 27 | de novo | M | 2005/12/24 | TSC1 | Frame shift | 20 | c.2509_2512delAACA | p.Asn837Valfs*11 |
| 28 | de novo | M | 2010/12/20 | TSC1 | Nonsense | 15 | c.1525C>T | p.Arg509* |
| 29 | de novo | M | 2011/10/8 | TSC1 | Frame shift | 17 | c.2111_2118delATGAGCGT | p.Y704FfsX |
| 30 | de novo | F | 2012/6/30 | TSC1 | Nonsense | 17 | c.2074C>T | p.Arg692* |
| 31 | de novo | F | 2011/10/15 | TSC1 | Frame shift | 15 | c.1612_1613del | p.Ser538Leufs*8 |
| 32 | de novo | F | 2008/12/22 | TSC2 | Splicing | 29 | c.3397+1G>A |  |
| 33 | de novo | M | 2014/8/5 | TSC2 | Missense | 39 | c.5012T>G | p.V1671G |
| 34 | de novo | F | 2014/6/10 | TSC2 | Frame shift | 16 | c.1667_1668insCTTG | p.Glu558Leufs*32 |
| 35 | de novo | M | 2007/9/11 | TSC2 | Frame shift | 38 | c.4944dupT | p.V1649Cfs*3 |
| 36 | de novo | F | 2011/10/22 | TSC2 | Nonsense | 18 | c.1874C>G | p.S625* |
| 37 | maternal | M | 2014/1/13 | TSC2 | Nonsense | 22 | c.2380C>T | p.Gln794* |
| 38 | de novo | M | 2013/9/30 | TSC2 | Splicing | 11 | c.976-1G>A |  |
| 39 | maternal | M | 2016/8/16 | TSC2 | Frame shift | 25 | c.2802_2817del | p.R934Sfs*7 |
| 40 | de novo | M | 2017/9/25 | TSC2 | Small deletion | 41 | c.5238_5255del | p.His1746_Arg1751del |
| 41 | de novo | M | 2015/1/25 | TSC2 | Frame shift | 16 | c.1688delC | p.V564Sfs*133 |
| 42 | paternal | F | 2016/10/25 | TSC2 | Missense | 17 | c.1789C>T | p.His597Tyr |
| 43 | de novo | F | 2009/11/12 | TSC2 | Large deletion | 40 | EX41del |  |
| 44 | de novo | M | 2011/11/19 | TSC2 | Large deletion | 2 | E2-E42del |  |
| 45 | maternal | F | 2011/7/20 | TSC2 | Missense | 22 | c.2540T>C | p.Leu847Pro |
| 46 | paternal | F | 2015/9/15 | TSC2 | Nonsense | 16 | c.1639G>T | p.547* |
| 47 | paternal | M | 2013/4/22 | TSC2 | Frame shift | 6 | c.482_485dupTGTC | p.Glu1602Valfs*27 |
| 48 | de novo | F | 2013/9/9 | TSC2 | Nonsense | 35 | c.4507C>T | p.Gln1503* |
| 49 | de novo | M | 2013/3/25 | TSC2 | Splicing | 21 | c.2355+2_2355+5del |  |
| 50 | de novo | M | 2004/6/23 | TSC2 | Frame shift | 8 | c.746dupT | p.K250Qfs*87 |
| 51 | de novo | F | 2011/9/14 | TSC2 | Frame shift | 34 | c.4123_4024 | P.S1375CfsX15 |
| 52 | de novo | M | 2014/2/6 | TSC2 | Small deletion | 41 | c.5238_5255del | p.His1746_Arg1751del |
| 53 | de novo | F | 2008/1/7 | TSC2 | Missense | 16 | c.1700T>G | p.L567R |
| 54 | de novo | M | 2010/4/3 | TSC2 | Nonsense | 6 | c.598C>T | p.Q200* |
| 55 | de novo | M | 2015/6/3 | TSC2 | Large deletion | 31 | EX31-42del |  |
| 56 | de novo | M | 2014/8/31 | TSC2 | Splicing | 40 | c.5069-1G>A |  |
| 57 | paternal | M | 2017/7/9 | TSC2 | Small deletion | 4 | c.277-301del | p.R93Cfs*4 |
| 58 | de novo | M | 2012/12/17 | TSC2 | Nonsense | 34 | c.4027G>T | p.Glu1343* |
| 59 | maternal | M | 2013/11/8 | TSC2 | Frame shift | 34 | c.4033_4034del | p.Lys1345Valfs*68 |
| 60 | de novo | M | 2016/10/14 | TSC2 | Splicing | 38 | c.4850-2A>G |  |
| 61 | de novo | F | 2004/7/15 | TSC2 | Missense | 40 | c.5126C>T | p.Pro1709Leu |
| 62 | de novo | F | 2014/2/21 | TSC2 | Frame shift | 41 | c.5197_5212del | p.(Thr1733Profs*88) |
| 63 | de novo | F | 2016/11/23 | TSC2 | Missense | 17 | c.1832G>A | p.R611Q |
| 64 | paternal | F | 2015/12/21 | TSC2 | Splicing | 11 | c.1119G>A |  |
| 65 | de novo | F | 2016/4/15 | TSC2 | Nonsense | 21 | c.2251C>T | p.R751* |
| 66 | paternal | F | 2005/11/27 | TSC2 | Nonsense | 34 | c.4397C>A | p.S1466* |
| 67 | de novo | M | 2013/5/20 | TSC2 | Nonsense | 29 | c.3395C>A | p.Ser1132* |
| 68 | de novo | M | 2014/10/21 | TSC2 | Missense | 27 | c.3073A>C | p.T1025P |
| 69 | de novo | F | 2013/5/4 | TSC2 | Missense | 29 | c.3598C>T | p.Arg1200Trp |
| 70 | maternal | M | 2014/3/16 | TSC2 | Small deletion | 41 | c.5238_5255del | p.His1746_Arg1751del |
| 71 | de novo | F | 2010/4/13 | TSC2 | Missense | 9 | c.825C>A | p.Asn275Lys |
| 72 | de novo | F | 2009/12/26 | TSC2 | Nonsense | 4 | c.268C>T | p.Q90* |
| 73 | de novo | F | 2015/11/2 | TSC2 | Nonsense | 11 | c.1117C>T | p.Q373* |
| 74 | de novo | F | 2015/11/3 | TSC2 | Frame shift | 15 | c.1501-1502delCA | p.D501Pfs*85 |
| 75 | de novo | M | 2013/10/8 | TSC2 | Missense | 41 | c.5227C>T | p.R1743W |
| 76 | paternal | F | 2013/6/12 | TSC2 | Missense | 34 | c.4110C>T | p.P1370P |
| 77 | de novo | F | 2003/10/15 | TSC2 | Nonsense | 34 | c.4129C>T | p.Gln1377* |
| 78 | de novo | M | 2009/7/10 | TSC2 | Missense | 38 | c.4952A>C | p.Asn1651Thr |
| 79 | de novo | M | 2011/6/17 | TSC2 | Small deletion | 41 | c.5238_5255del | p.His1746_Arg1751del |
| 80 | de novo | M | 2011/12/11 | TSC2 | Frame shift | 31 | c.3682delC | p.L1228cfsX97 |
| 81 | de novo | M | 2017/12/20 | TSC2 | Nonsense | 4 | c.268C>T | p.Gln90* |
| 82 | de novo | M | 2011/5/6 | TSC2 | Small deletion | 42 | c.5422_5423del | p.*1808Argext*33 |
| 83 | de novo | M | 2016/8/13 | TSC2 | Missense | 39 | c.5024C>T | p.Pro1675Leu |
| 84 | de novo | F | 2014/4/28 | TSC2 | Frame shift | 38 | c.4909_4910delAA | p.(Lys1637Glufs*15) |
| 85 | de novo | M | 2011/1/17 | TSC2 | Frame shift | 41 | c.5166_5181del | p.Ser1723Alafs*98 |
| 86 | paternal | M | 2012/12/28 | TSC2 | Nonsense | 30 | c.3412C>T | p.R1138* |
| 87 | de novo | F | 2017/11/23 | TSC2 | Frame shift | 19 | c.2052delG | p.Y686Tfs*11 |
| 88 | de novo | F | 2013/5/23 | TSC2 | Missense | 30 | c.3421G>A | p.Ala1141Thr |
| 89 | de novo | M | 2016/7/5 | TSC2 | Splicing | 7 | c.600-1G>A |  |
| 90 | paternal | F | 2016/10/28 | TSC2 | Missense | 41 | c.5227C>T | p.R1743W |
| 91 | maternal | M | 2012/7/5 | TSC2 | Missense | 41 | c.5228G>A | p.Arg1743Gln |
| 92 | de novo | F | 2008/10/5 | TSC2 | Nonsense | 10 | c.932_936 del CTCTC | p.S311* |
| 93 | de novo | M | 2012/6/14 | TSC2 | Nonsense | 30 | c.3532C>T | p.Gln1178* |
| 94 | de novo | F | 2013/8/21 | TSC2 | Frame shift | 8 | c.706dupC | p.L236Pfs*102 |
| 95 | de novo | F | 2010/12/5 | TSC2 | Large deletion | 2 | EX2-42del |  |
| 96 | de novo | M | 2003/12/28 | TSC2 | Missense | 8 | c.656T>C | p.Leu219Pro |
| 97 | de novo | M | 2007/4/18 | TSC2 | Splicing | 15 | c.1599G>A |  |
| 98 | de novo | F | 2011/10/21 | TSC2 | Missense | 30 | c.3598C>T | p.Arg1200Trp |
| 99 | de novo | M | 2017/9/23 | TSC2 | Large deletion | 5 | EX5del |  |
| 100 | de novo | F | 2017/9/7 | TSC2 | Frame shift | 4 | c.292delC | p.R98Gfs*7 |
| 101 | de novo | F | 2018/8/29 | TSC2 | Splicing | 22 | c.2545+5G>C |  |
| 102 | de novo | F | 2011/7/14 | TSC2 | Nonsense | 9 | c.822C>A | p.Y274Y* |
| 103 | de novo | M | 2011/4/27 | TSC2 | Frame shift | 41 | c.5173del | p.(Val1725Cysfs*101) |
| 104 | de novo | F | 2013/2/10 | TSC2 | Frame shift | 30 | c.3607delA | p.Thr1203Glnfs*7 |
| 105 | de novo | M | 2013/11/24 | TSC2 | Nonsense | 3 | c.223G>T | p.E75E* |
| 106 | de novo | F | 2011/4/25 | TSC2 | Frame shift | 34 | c.4013_4014del | p.Ser1338Cysfs*75 |
| 107 | de novo | F | 2018/2/24 | TSC2 | Missense | 38 | c.4943T>C | p.Ile1648Thr |
| 108 | de novo | F | 2016/10/16 | TSC2 | Missense | 37 | c.4709G>C | p.R1570T |
| 109 | de novo | F | 2014/12/13 | TSC2 | Frame shift | 41 | c.5281_5296del16 | p.Ser1761Leufs*60 |
| 110 | de novo | F | 2003/12/20 | TSC2 | Nonsense | 37 | c.4804-4805insTACCTGTCCTG | p.Glu1602Valfs*27 |
| 111 | de novo | F | 2016/1/20 | TSC2 | Frame shift | 16 | c.1660_1663dup | p.A555Vfs*33 |
| 112 | de novo | M | 2015/4/5 | TSC2 | Nonsense | 31 | c.3624G>A | p.W1208* |
| 113 | de novo | F | 2007/3/25 | TSC2 | Missense | 17 | c.1831C>T | p.Arg611Trp |
| 114 | de novo | M | 2012/11/23 | TSC2 | Frame shift | 12 | c.1350_1353delGAGA | p.E450Dfs*33 |
| 115 | de novo | F | 2015/1/29 | TSC2 | Missense | 30 | c.3489C>T | p.A1163A |
| 116 | de novo | F | 2009/11/14 | TSC2 | Frame shift | 34 | c.4439_4439insA | p.Leu1480Tyrfs*44 |
| 117 | de novo | F | 2017/12/26 | TSC2 | Small deletion | 41 | c.5224_5241del | p.Ala1742_Ile1747del |
| 118 | de novo | M | 2014/8/20 | TSC2 | Frame shift | 25 | c.2766delG, p.Leu922PhefsX26 | p.Leu922Phefs*26 |
| 119 | de novo | F | 2012/4/11 | TSC2 | Splicing | 30 | c.3610+1G>A |  |
| 120 | de novo | M | 2008/2/5 | TSC2 | Splicing | 6 | c.599+4A>G |  |
| 121 | de novo | F | 2003/7/5 | TSC2 | Nonsense | 34 | c.4183C>T | p.Q1395* |
| 122 | de novo | M | 2007/6/29 | TSC2 | Missense | 41 | c.5228G>A | p.R1743Q |
| 123 | de novo | F | 2010/12/15 | TSC2 | Nonsense | 19 | c.2058C>A | p.Y686* |
| 124 | de novo | F | 2007/8/30 | TSC2 | Frame shift | 41 | c.5195_5196insTGCA | p.Thr1733Alafs*43 |
| 125 | de novo | M | 2008/5/20 | TSC2 | Missense | 13 | c.1361G>T | p.R454RM |
| 126 | de novo | F | 2017/6/26 | TSC2 | Small deletion | 38 | c.4909_4911delAAG | p.Lys1638del |
| 127 | de novo | F | 2012/9/26 | TSC2 | Missense | 37 | c.4720T>G | p.(Phe1574Val) |
| 128 | de novo | M | 2003/5/1 | TSC2 | Deletion-insertion | 31 | c.3813_3814delinsTGCCTCGCTCCAACAC |  |
| 129 | de novo | F | 2003/9/3 | TSC2 | Splicing | 25 | c.2743-2A>G |  |
| 130 | de novo | M | 2013/1/22 | TSC2 | Nonsense | 33 | c.4174C>T | p.Gln1392* |
| 131 | de novo | M | 2008/9/2 | TSC2 | Splicing | 21 | c.2221-2A>G |  |
| 132 | de novo | M | 2015/2/16 | TSC2 | Missense | 39 | c.5045T>C | p.Leu1682Pro |
| 133 | de novo | M | 2017/3/14 | TSC2 | Splicing | 21 | c.2221-1G>C |  |
| 134 | de novo | F | 2006/8/28 | TSC2 | Nonsense | 30 | c.3582G>A | p.W1194* |
| 135 | de novo | M | 2003/9/2 | TSC2 | Nonsense | 14 | c.1372C>T | p.Arg458* |
| 136 | de novo | F | 2005/12/14 | TSC2 | Splicing | 21 | c.2221-2A>G |  |
| 137 | de novo | M | 2015/7/14 | TSC2 | Nonsense | 34 | c.4024C>T | p.Gln1342* |
| 138 | de novo | M | 2005/10/12 | TSC2 | Nonsense | 41 | c.5220G>A | p.1740* |
| 139 | de novo | M | 2015/11/22 | TSC2 | Splicing | 13 | c.1258-1G>A |  |
| 140 | de novo | F | 2009/2/26 | TSC2 | Nonsense | 34 | c.4048G>T | p.Glu1350* |
| 141 | de novo | M | 2012/7/9 | TSC2 | Frame shift | 34 | c.4351dupC | p.(Arg1451Profs*73) |
| 142 | paternal | F | 2017/8/25 | TSC2 | Splicing | 26 | c.2838-122G>A | p.Ser946Argfs*6 |
| 143 | de novo | F | 2015/7/20 | TSC2 | Frame shift | 2 | c.133_136delCTGA | p.Leu45Glufs*3 |
| 144 | de novo | M | 2012/3/2 | TSC2 | Missense | 39 | c.5024C>T | p.(Pro1675Leu) |
| 145 | paternal | F | 2017/11/23 | TSC2 | Nonsense | 34 | c.4355C>A | p.1452* |
| 146 | de novo | M | 2015/6/2 | TSC2 | Deletion-insertion | 11 | c.1033_1035delinsTTA | p.L345L |
| 147 | de novo | F | 2013/12/26 | TSC2 | Missense | 19 | c.2088C>G | p.C696CW |
| 148 | paternal | F | 2010/8/9 | TSC2 | Missense | 10 | c.899G>T | p.G300GV |
| 149 | paternal | F | 2015/1/4 | TSC2 | Nonsense | 2 | c.136A>T | p.R46R* |
| 150 | de novo | F | 2014/11/21 | TSC2 | Large deletion | 21 | c.2346_2359del |  |
| 151 | de novo | M | 2004/3/10 | TSC2 | Large deletion | 2 | EX2-42del |  |
| 152 | de novo | M | 2010/1/21 | NMI |  |  |  |  |
| 153 | de novo | F | 2010/7/31 | NMI |  |  |  |  |
| 154 | de novo | M | 2015/1/26 | NMI |  |  |  |  |
| 155 | de novo | F | 2013/12/24 | NMI |  |  |  |  |
| 156 | de novo | M | 2014/11/18 | NMI |  |  |  |  |
| 157 | de novo | M | 2011/10/10 | NMI |  |  |  |  |
| 158 | de novo | F | 2011/12/28 | NMI |  |  |  |  |
| 159 | de novo | F | 2017/7/18 | NMI |  |  |  |  |
| 160 | de novo | F | 2016/7/28 | NMI |  |  |  |  |
| 161 | de novo | M | 2013/1/10 | NMI |  |  |  |  |
| 162 | de novo | F | 2016/7/15 | NMI |  |  |  |  |
| 163 | maternal | F | 2016/12/16 | NMI |  |  |  |  |
| 164 | de novo | F | 2012/10/21 | NMI |  |  |  |  |
| 165 | de novo | M | 2008/12/5 | NMI |  |  |  |  |
| 166 | de novo | M | 2014/2/14 | NMI |  |  |  |  |
| 167 | de novo | F | 2008/7/3 | NMI |  |  |  |  |
| 168 | de novo | M | 2003/8/7 | NMI |  |  |  |  |
| 169 | de novo | M | 2003/6/18 | NMI |  |  |  |  |
| 170 | de novo | F | 2016/3/11 | NMI |  |  |  |  |
| 171 | de novo | M | 2016/10/16 | NMI |  |  |  |  |
| 172 | de novo | F | 2005/4/16 | NMI |  |  |  |  |
| 173 | de novo | F | 2009/1/23 | NMI |  |  |  |  |
| 174 | de novo | M | 2015/8/25 | NMI |  |  |  |  |
